# Organization of GABAergic Synaptic Circuits in the Rat Ventral Tegmental Area

**DOI:** 10.1371/journal.pone.0046250

**Published:** 2012-10-08

**Authors:** Alessandro Ciccarelli, Arianna Calza, Patrizia Panzanelli, Alessandra Concas, Maurizio Giustetto, Marco Sassoè-Pognetto

**Affiliations:** 1 Department of Anatomy, Pharmacology and Forensic Medicine, University of Turin, and National Institute of Neuroscience-Italy, Torino, Italy; 2 Department of Experimental Biology and Center of Excellence for the Neurobiology of Dependence, University of Cagliari, Cagliari, Italy; Institute for Interdisciplinary Neuroscience, France

## Abstract

The ventral tegmental area (VTA) is widely implicated in drug addiction and other psychiatric disorders. This brain region is densely populated by dopaminergic (DA) neurons and also contains a sparse population of γ-aminobutyric acid (GABA)ergic cells that regulate the activity of the principal neurons. Therefore, an in-depth knowledge of the organization of VTA GABAergic circuits and of the plasticity induced by drug consumption is essential for understanding the mechanisms by which drugs induce stable changes in brain reward circuits. Using immunohistochemistry, we provide a detailed description of the localization of major GABA_A_ and GABA_B_ receptor subunits in the rat VTA. We show that DA and GABAergic cells express both GABA_A_ and GABA_B_ receptors. However VTA neurons differ considerably in the expression of GABA_A_ receptor subunits, as the α1 subunit is associated predominantly with non-DA cells, whereas the α3 subunit is present at low levels in both types of VTA neurons. Using an unbiased stereological method, we then demonstrate that α1-positive elements represent only a fraction of non-DA neurons and that the ratio of DA and non-DA cells is quite variable throughout the rostro-caudal extent of the VTA. Interestingly, DA and non-DA cells receive a similar density of perisomatic synapses, whereas axo-dendritic synapses are significantly more abundant in non-DA cells, indicating that local interneurons receive prominent GABAergic inhibition. These findings reveal a differential expression of GABA receptor subtypes in the two major categories of VTA neurons and provide an anatomical basis for interpreting the plasticity of inhibitory circuits induced by drug exposure.

## Introduction

The ventral tegmental area (VTA) is a midbrain region critically involved in natural reward and drug addiction [1], [2]. Dopamine (DA)-releasing neurons of the VTA send projections to limbic forebrain structures, including the prefrontal cortex, amygdala and nucleus accumbens. All addictive drugs studied so far exert their actions by increasing DA levels in the VTA target regions [3]. In addition, addictive drugs trigger synaptic plasticity in the VTA, leading to persistent modifications of neural circuits that are believed to underlie addictive behaviours [4]–[8].

In addition to DA cells, the VTA is populated by GABAergic neurons that provide an important regulatory influence over the activity of DA neurons, and also send projections to a variety of brain regions [9]–[13]. A population of GABAergic cells appears to be concentrated in the caudalmost tier of the VTA, the so-called tail of the VTA [14]. These neurons are activated by exposure to psychostimulants and to other drugs [14]–[17], and send GABAergic projections to the VTA and the substantia nigra pars compacta [18], [19]. Moreover, a recent optogenetic study demonstrated that a significant number of GABAergic, medium spiny neurons of the nucleus accumbens project back to the VTA, where they target non-DA neurons [20]. Therefore, it appears that different types of VTA and non-VTA neurons provide GABAergic inhibition of DA cells.

There is accumulating evidence that changes in GABAergic inhibition contribute to circuit modifications induced by several types of drugs of abuse. Specifically, opioids, cannabinoids, and γ-hydroxybutyrate affect the excitability of DA cells by suppressing GABA release from VTA interneurons [3], [21]–[27]. Notably, chronic exposure to opiates can dynamically regulate GABA_A_R signaling in neurons of the VTA and direct VTA output through separate brain reward systems [28]. Similarly cocaine, a drug that mainly affects dopamine transporters, decreases GABAergic inhibition and facilitates LTP in VTA neurons [5]. It has also been reported that benzodiazepines, that are allosteric modulators of GABA_A_Rs widely used in the treatment of anxiety, insomnia and seizures [29], increase dopamine levels and cause drug reinforcement through disinhibition of DA cells [30], [31]. Long-term exposure to benzodiazepines results in tolerance and dependence, which increase their potential for abuse and limit their clinical utility [32], [33]. Interestingly, application of the GABA_A_R agonist muscimol in the VTA can either increase or decrease DA levels in the nucleus accumbens, likely reflecting dose-dependent effects on GABA_A_Rs expressed by DA cells and GABAergic interneurons [9]–[11], [23].

The present study was undertaken to ameliorate our understanding of the organization of GABAergic synaptic circuits of the rat VTA. Using immunohistochemistry, we show that there is a largely differential expression of GABA_A_Rs containing either the α1 or the α3 subunit in DA and non-DA cells, whereas GABA_B_Rs are expressed in both cell types. We also compare GABAergic innervation in DA and non-DA cells, and we provide a quantitative estimate of these two neuronal populations using unbiased stereology with an optical disector.

## Materials and Methods

### Tissue preparation and immunohistochemistry

The experiments described in this study were performed on adult male Sprague–Dawley CD rats raised under standard laboratory conditions with unrestricted access to food and water. The experimental procedures were approved by the Italian Ministry of Health and by the Bioethic Committee of Turin University in accordance with national (Legislative Decree 116/92 and law n. 413/1993) and international (Directive 86/609/EEC and the recommendation 2007/526/EC from European community) laws and policies. Animals were anaesthetised with an intraperitoneal injection of chloral hydrate and transcardially perfused with ice cold formaldehyde (4% in 0.1 M phosphate buffer – PB, pH 7.4). After perfusion, the brains were dissected and kept in the same fixative at 4°C overnight. After several washes in PB, brains were cryoprotected by immersion in 10%, 20%, and 30% sucrose solutions, cut in 16-µm or 30-µm sections with a cryostat and stored at −20°C. In order to analyze the subcellular localization of GABA_A_R subunits we also used a brief-fixation protocol that has been optimized for in situ detection of postsynaptic molecules [34]. Briefly, the rat midbrain was cut manually in coronal slices (∼1 mm) that were fixed by immersion in 4% formaldehyde for 30 minutes (for details see ref. [35]). The sections were then cryoprotected in sucrose (10%, 20% and 30%) and sectioned with a cryostat at 16 µm.

For immunofluorescence, sections were first blocked with a solution containing 10% normal goat serum (NGS) and 0.05% Triton X-100 in phosphate-buffered saline (PBS; pH 7.4) and then incubated overnight with combinations of two or three primary antibodies raised in different species (see Table 1 for antibody specification). The sections were then rinsed in PBS and incubated with the appropriate secondary antibodies raised either in goat or in donkey and conjugated to Alexa 488 (Molecular Probes, Eugene, Oregon) or the cyanines Cy3 and Cy5 (Jackson ImmunoResearch, West Grove, PA). Finally, the sections were rinsed and coverslipped with Dako fluorescence mounting medium (Dako Italia, Italy).

**Table 1:** Primary antibodies used in this study.

Antibody	Immunogen	Source – Cat. Number - Species	Dilution
Tyrosine hydroxilase (TH)	TH purified from rat PC12 cells	Immunostar, no. 22941mouse monoclonal	1∶4000
GABA_A_R α1	Rat N-terminal peptide amino acids 1–16	H. Mohler and J.-M. Fritschy (Institute of Pharmacology and Toxicology, University of Zurich, Switzerland)Guinea pig polyclonal	1∶5000
GABA_A_R α1	Rat N-terminal peptide amino acids 1–16	H. Mohler and J.-M. FritschyRabbit polyclonal	1∶5000
GABA_A_R α3	Rat N-terminal peptide amino acids 1–15	H. Mohler and J.-M. FritschyGuinea pig polyclonal	1∶4000
NeuN	Purified cell nuclei from mouse brain	Chemicon/Millipore, code MAB377Mouse monoclonal	1∶5000
GABA_B_R1	Synthetic peptide from rat GABA_B_ receptor	Chemicon, code AB1531Guinea pig polyclonal	1∶4000
Vesicular GABA transporter (VGAT)	Synthetic peptide AEPPVEGDIHYQR (aa 75–87 in rat) coupled to keyhole limpet hemocyanin via an added N- terminalcysteine	Synaptic Systems, no. 131003Rabbit polyclonal, affinity purified	1∶3000

### Microscopy and data analysis

For cell density quantification six coronal sections (30 µm) of rat midbrain derived from a 1∶6 series were assigned to three VTA rostrocaudal levels: rostral VTA (bregma −5.00 to −5.40 mm), middle VTA (bregma −5.40 to −5.80 mm) and caudal VTA (bregma −5.80 to −6.20 mm). Quantification of cell densities was done at these three rostrocaudal levels in the major subnuclei of the VTA (PBP, PN, IF, RLi, CLi), according to Paxinos and Watson [36]. For each rostrocaudal level, the sampling regions were eight in the PBP and PN, and four in the other VTA subnuclei (RLi, IF, and CL). Each sampling region was represented by a Z-stack composed of four confocal sections (1024×1024 pixels corresponding to 230 µm×230 µm) spaced 2.50 µm. Confocal images were acquired with a laser scanning confocal microscope (Zeiss LSM5 Pascal) with a 40× oil-immersion objective using the multi-track mode. We first calculated the volumetric density of NeuN-positive cells by counting cells that were present in the second (lookup) section but not in the fourth (reference) section of each confocal stack. Subsequently, we assessed the degree of colocalization of NeuN with TH and GABA_A_R α1 in the counted cells.

To calculate the density of VGAT boutons contacting TH- and α1-positive cells, we acquired confocal images at high resolution with a 100× oil-immersion objective (1.4 NA) and the pinhole set at 1 Airy unit. Each confocal image was composed by four optical sections spaced 0.37 µm. A minimum of ten pictures derived from the rostral and the caudal VTA were used. On every cell we estimated the linear density of synaptic appositions by calculating manually the number of clusters in direct contact with the cell body or the dendritic profiles. All analyses were done with the NIH ImageJ software.

### Statistical Analysis

Statistical analysis was done by two-way ANOVA using SPSS 12.0 software (SPSS Inc., Chicago, IL). Significance threshold was set at p<0.05. All data are presented as means ± SEM.

## Results

### The GABA_A_R α1 subunit is mainly expressed by non-DA cells whereas the α3 subunit is expressed by both DA and non-DA cells

We initially labeled VTA sections with antibodies directed against different GABA_A_R subunits (α1, α2, α3, α4, α5, δ). We observed prominent labeling for the GABA_A_R α1 subunit and moderate labeling for GABA_A_R α3, whereas the other subunits were undetectable or expressed at very low levels (not shown). With few exceptions, these observations are in good agreement with previous immunohistochemical reports [37]–[39], and indicate that GABA_A_Rs containing the α1 and/or α3 subunits are predominant in the VTA.

**Figure 1:**
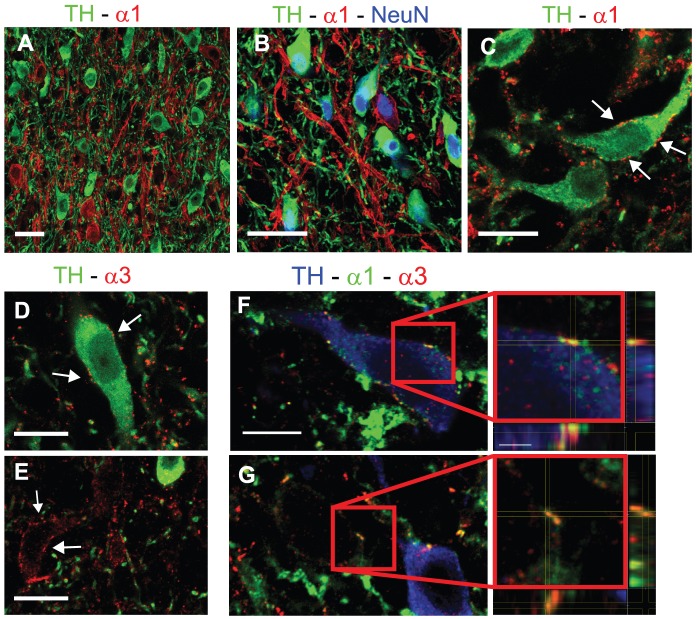
Differential distribution of α1- and α3-GABA_A_Rs in DA and non-DA cells. (**A**) Representative image showing that labeling for GABA_A_R α1 is generally not associated with TH-positive neurons. (**B**) Triple labeling showing that TH-positive and α1-positive neurons are labeled for NeuN. (**C**) Double labeling for TH and GABA_A_R α1 in a section processed with the brief-fixation protocol. Arrows indicate α1-immunolabeled clusters at the surface of a TH-positive neuron. (**D,E**) Double labeling for GABA_A_R α3 and TH on briefly-fixed tissue. Labeling for the α3 subunit (arrows) is present both in TH-positive (**D**) and TH-negative cells (**E**). (**F,G**) Confocal images from triple-labeled sections showing co-localization of the α1 and α3 subunits in TH-positive (**F**) and TH-negative cells (**G**). The boxed areas are enlarged and shown as tridimensional projections. Scale bars: A,B = 30 µm. C–E = 12 µm. F,G = 10 µm (inset = 2 µm).

We therefore investigated the expression of these GABA_A_R subunits in relation to the two major neuronal populations of the VTA, DA cells and non-DA cells. Using triple immunofluorescence for TH, GABA_A_R α1 and the neuronal marker NeuN in perfused tissue, we found that the α1 subunit was mainly expressed by non-DA cells (Fig. 1A,B), although occasionally we observed TH-positive elements that were also labeled for GABA_A_R α1. We also used a more sensitive immunofluorescence protocol (see Materials and Methods), to determine whether DA cells express the α1 subunit. Using this approach, found that α1-positive clusters decorated only occasionally the surface of TH-positive cells (Fig. 1C), although in this case we could not estimate the number of DA cells expressing GABA_A_R α1, nor the density of GABA_A_R α1-positive clusters on individual neurons. These data are in agreement with previous observations in the mouse VTA [31], and indicate that TH-positive DA neurons do not usually contain α1 GABA_A_Rs.

We then analyzed the distribution of the GABA_A_R α3 subunit. Given the low fluorescence signals in perfused tissue (not shown), we labeled sections processed according to the weak-fixation protocol, that affords higher sensitivity. Double labelling for GABA_A_R α3 and TH showed that the α3 subunit was expressed by DA cells (Fig. 1D). However, there were also cases in which TH-negative neurons were decorated by α3-positive clusters (Fig. 1E), suggesting that the α3 subunit was expressed also by a subset of non-DA cells. Triple labelling for TH, GABA_A_R α1 and GABA_A_R α3 showed that the α1 and α3 subunits co-existed at the level of individual clusters in both DA and non-DA cells (Fig. 1F,G). Also in this case, we did not perform a quantitative analysis of colocalization due to the inherent difficulties associated with this kind of examination. However, these data reveal a complex subunit composition of GABA_A_Rs expressed by VTA neurons.

### DA and non-DA cells in the VTA

**Figure 2:**
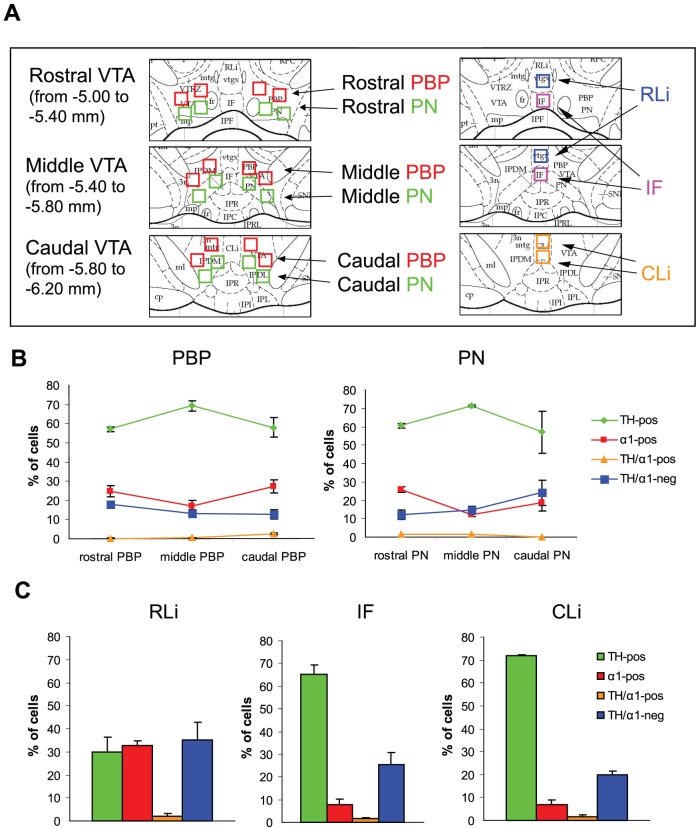
Quantification of TH-positive and α1-positive neurons in the VTA. (**A**) Schematic diagrams of the VTA subnuclei at rostral (Bregma −5.0 to −5.4 mm), middle (Bregma −5.4 to −5.8 mm) and caudal (Bregma −5.8 to −6.2 mm) VTA levels as shown in coronal sections (from Paxinos and Watson, ref. [36]). The boxed areas indicate the position of the sampling fields utilized for cell density quantification (230×230 µm). PBP, parabrachial nucleus; PN, paranigral nucleus; IF, interfascicular subnucleus; RLi, rostral linear nucleus; CLi, caudal linear nucleus. (**B**) Relative abundance of VTA neurons in the parabrachial (PBP) and paranigral (PN) nuclei at different rostro-caudal levels. Four neuronal poulations of NeuN-positive cells were identified: TH-positive (green), α1-positive (red), TH/α1-positive (blue) and TH/α1-negative (yellow). (**C**) Relative abundance of the same neuronal populations in the medial VTA subnulcei. Data represent the mean ± SEM of 3 rats.

We next performed a cell density analysis in the main subnuclei of the VTA (Fig. 2A). We applied a stereological method with an optical disector in sections triple labeled for TH, GABA_A_R α1 and NeuN to determine the volumetric density of NeuN-positive neurons, and we then determined the ratio of NeuN-positive cells that were also labeled for TH and/or the α1 subunit (Table 2). In the more lateral subnuclei of the VTA (PBP and PN), DA cells accounted for approximately 60–70% of the total neuronal population, with slightly higher values in the middle VTA compared with the rostral and caudal subdivisions (Fig. 2B). In contrast, the percentage of α1-positive cells ranged from 17 to 25, with a rostro-caudal trend opposite to that of DA neurons, implying that the TH/α1 ratio was higher in the middle VTA and then decreased at more rostral and caudal levels (Fig. 2B). Interestingly, a substantial number of NeuN-positive neurons was labeled for neither TH nor GABA_A_R α1 (Fig. 2B and Table 2), suggesting that non-DA neurons can be subdivided in two distinct subgroups based on whether they express or not the α1 subunit.

**Table 2:** Stereological estimate of neuronal populations of the rat VTA.

Density (cells/mm^3^)×10^3^				
	TH-positive	α1-positive	TH-negativeα1-negative	TH-positiveα1-positive
Rostral PBP	12.5±1.3	5.4±0.6	4.0±0.6	0.1±0.1
Middle PBP	8.2±1.2	2.0±0.4	1.7±0.5	0.1±0.0
Caudal PBP	6.6±0.6	3.1±0.4	1.4±0.3	0.3±0.1
Rostral PN	12.1±2.5	5.1±1.0	2.6±1.1	0.3±0.1
Middle PN	15.3±1.1	2.7±0.5	3.1±0.1	0.3±0.1
Caudal PN	13.2±2.9	4.4±1.4	5.7±2.0	0.0±0.0
RLi	5.6±1.3	6.3±0.9	6.8±1.7	0.4±0.2
IF	25.0±3.1	2.9±0.8	9.7±2.0	0.7±0.2
CLi	25.5±2.4	2.6±0.9	6.9±0.4	0.5±0.3
Rostral VTA	10.4±0.5 (43%)	6.5±0.8 (26.9%)	7.1±0.8 (29.3%)	0.2±0.1 (0.8%)
Middle VTA	16.3±1.4 (70.3%)	2.1±0.6 (9.1%)	4.4±1.2 (18.9%)	0.4±0.2 (1.7%)
Caudal VTA	15.1±0.7 (64.3%)	3.4±0.9 (14.4%)	4.7±0.7 (20%)	0.3±0.1 (1.3%)
**Total VTA**	13.9±0.6 (58.9%)	4.0±0.1 (16.9%)	5.4±0.8 (22.9%)	0.3±0.1 (1.3%)

The values of rostral, middle and caudal VTA have been obtained averaging the values of all the subnuclei at each individual rostro-caudal level. Percentage values are referred to all NeuN-positive cells counted in the corresponding region. Values are means ± S.E.M. of 3 rats.

The situation in the medial VTA subnuclei was more heterogeneous. A particularly low density of α1-positive cells was observed in the IF and CLi, whereas the RLi was characterized by a rather low quantity of TH-positive neurons and an unusually large percentage of α1-positive cells as well as cells that were neither α1-positive nor TH-positive (Fig. 2C and Table 2). In all the VTA subregions analyzed the percentage of cells double-labeled for TH and GABA_A_R α1 was very low, as noted above.

### Organization of GABAergic inputs in DA cells and α1-positive cells

**Figure 3:**
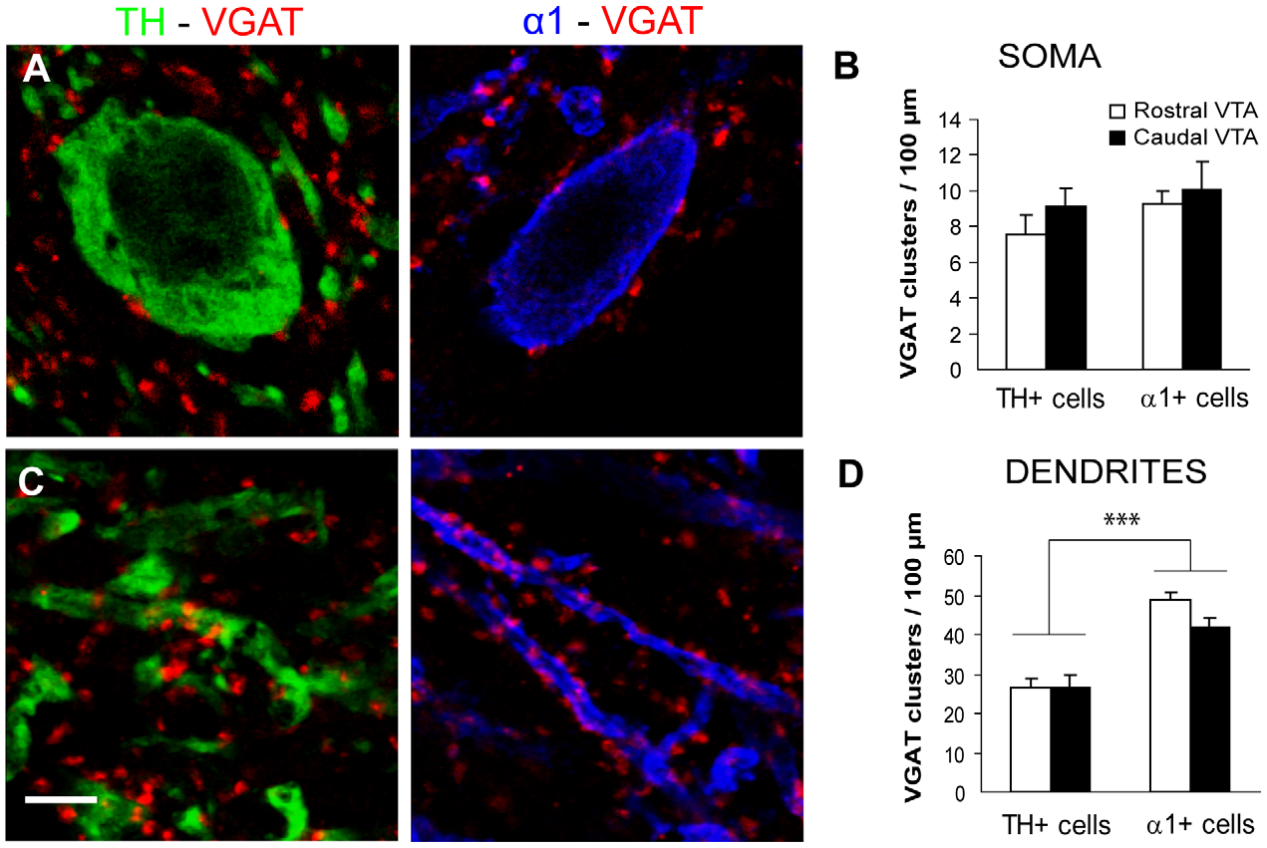
Perisomatic and axodendritic innervation of DA and non-DA cells. (**A**) Representative confocal images showing perisomatic innervation of TH-positive (left) and α1-positive cells (right) by GABAergic axonal boutons identified by VGAT immunoreactivity. The quantitative analysis (**B**) revealed no difference in the density of perisomatic VGAT clusters between the two cell populations (type of cell, F_(1,69)_:1.505, p = 0.224) as well as between the rostral and caudal VTA (VTA subregion, F_(1,69)_:1.175, p = 0.282; two-way ANOVA, n = 15–22 cells per group). (**C**) VGAT-positive boutons contact dendritic profiles belonging to TH cells (left) or α1-positive cells (right). (**D**) The density of axo-dendritic innervation was significantly higher for α1-positive cells than for TH cells (type of cell, F_(1,119)_:64.409, p<0.001 ***), with no difference between rostral and caudal VTA (VTA subregion, F_(1,119)_:1.814, p = 0.181; two-way ANOVA, n = 23–38 fibers per group). Scale bar: 5 µm.

The results reported above suggest that DA cells express relatively low amounts of GABA_A_Rs as compared to non-DA cells. We therefore investigated whether this difference was correlated with a difference in the density of GABAergic inputs between the two cell types. We used an antiserum against the vesicular GABA transporter (VGAT) to identify GABAergic axon terminals contacting TH-positive and α1-positive cells in sections from perfused brains. We found a similar density of perisomatic contacts in the two cell populations, both in the rostral VTA and in the caudal VTA (Fig. 3A,B). In contrast, there was a higher density of axo-dendritic contacts in α1-positive cells compared with TH-positive cells in both VTA subregions (Fig. 3C,D). These data suggest that non-DA cells receive a higher amount of GABAergic synapses on their dendrites compared to DA cells.

### GABA_B_Rs are expressed by both DA cells and non-DA cells

**Figure 4:**
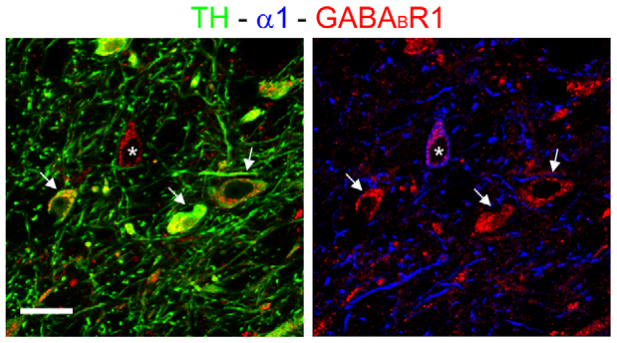
Ubiquitous expression of GABA_B_R1 in neurons of the VTA. Triple labeling showing the expression of GABA_B_R1 in both TH-positive cells (left, arrows) and α1-positive neurons (right, asterisk). Note that labeling for GABA_B_R1 extends into the dendritic profiles of TH-positive, but not α1-positive neurons. Scale bar: 30 µm.

We then used an antibody against the obligatory GABA_B_R1 subunit [40], [41] to investigate the expression of GABA_B_Rs in the two neuronal populations of the VTA. We found labeling for GABA_B_R1 in practically all cells that were positive for either TH or GABA_A_R α1 (Fig. 4). The immunoreactivity was localized in neuronal perikarya and also extended in some dendritic profiles that were TH-positive. In contrast, α1-positive dendrites were generally unlabeled for GABA_B_R1 (Fig. 4). These data indicate that GABA_B_Rs are expressed by both DA cells and non-DA cells, and suggest the existence of differences in subcellular distribution between these two cell populations.

## Discussion

In this study we show that there is a differential expression of GABA_A_R subtypes in DA and non-DA cells of the rat VTA. While GABA_A_Rs containing the α1 subunit are mainly expressed by non-DA cells, the α3 subunit is expressed at lower levels by both DA and non-DA cells. In addition, both populations of neurons appear to contain GABA_B_Rs. Our results are in agreement with a recent study of the mouse VTA, in which GABA_A_R α1 and GABA_A_R α3 were found to be largely segregated in TH-positive and GAD67-GFP-positive cells, respectively [31]. However, our study differs from the previous one mainly because we identified a population of NeuN-positive cells that were neither TH-positive nor α1-positive, suggesting that labeling for the α1 subunit identifies only a subset of non-DA neurons.

By combining labeling for TH, GABA_A_R α1 and NeuN, we estimated the ratio of DA and non-DA cells in different subnuclei of the VTA (Fig. 2). There have been several previous attempts to quantify the population of DA and non-DA cells in the VTA, however a comparison of the results is not simple due to the different methodologies and the different sampling procedures used in different studies. The overall density of DA cells reported here (Table 2) is in line with another investigation in which the density of TH-positive neurons was estimated using an unbiased optical fractionator [42]. Three main conclusions can be derived from the analysis of the data represented in Table 2. First, the ratio of DA and non-DA cells is quite variable throughout the VTA rostro-caudal extent. In particular, the density of TH-positive cells is considerably lower in the rostral VTA as compared with the middle and caudal subdivisions, a conclusion that is consistent with previous observations reporting greater numbers of DA neurons in more caudal subregions of the VTA [43]–[45]. It is likely that the relatively low density of TH-positive elements in the RLi contributed significantly to the uneven rostro-caudal distribution of DA neurons observed here. Second, non-DA cells account for 30–35% of neurons of the middle and caudal VTA, with even higher values (56%) in the rostral subdivision. Again, this estimate agrees quite well with previous reports according to which at least one third of VTA neurons are non-DA [43], [45], [46]. Finally, our results indicate that non-DA cells fall into α1-positive and α1-negative subtypes, which account for approximately 17% and 23% of the entire neuronal population of the VTA (Table 2). It is tempting to speculate that α1-positive and α1-negative cells may correspond to functionally distinct subtypes of non-DA neurons, including a population of so-called tertiary cells that have a distinct pharmacological profile [45], [47]. According to Cameron et al. [47], tertiary cells represent 27.4% of the neuronal population of the guinea pig VTA, a percentage that is close to the one reported here for non-DA α1-negative neurons. Further studies will have to evaluate the expression of the α1 subunit as a possible molecular signature for a specific population of VTA non-DA neurons.

The neurochemical identity of the two subtypes of non-DA cells identified in the present study remains poorly characterized. It is likely that most of the non-DA cells are GABAergic neurons. In fact, a quantitative study based on unbiased stereology reported that 35% of all cells in the rat VTA are GABAergic [46]. This is also consistent with the idea that the majority of VTA neurons are either DA or GABAergic [21], [48]. However, a recent investigation in GAD67-GFP mice showed that TH-positive neurons are five times more abundant than GAD-GFP cells [48]. The reasons for these discrepancies are unclear, but may simply reflect differences between mouse and rat, as well as differences in the VTA subregions analyzed in these studies. Glutamatergic neurons have also been identified in the VTA based on expression of the mRNA for the vesicular glutamate transporter VGlut2 [49], [50]. However, according to Nair-Roberts et al. [46], glutamatergic neurons account for only 2–3% of the total population of VTA neurons, and they appear to be mainly enriched in medial parts of the rostral VTA. Unfortunately, we are not aware of the existence of immunohistochemical markers that allow unambiguous identification of these glutamatergic neurons, therefore we could not directly assess the expression of the α1 subunit in this neuronal population.

The data discussed above are based on immunofluorescence labeling of sections obtained from perfused brains. This methods results in diffuse labeling of GABA_A_R subunits, which facilitates cell visualization and colocalization analysis. By using a more sensitive method, we found that α1-positive clusters were present occasionally also at the surface of TH-positive cells, suggesting that expression of α1-GABA_A_Rs is not uniquely restricted to non-DA cells. This is in agreement with observations in the mouse VTA [31], where diffuse labeling for the α1 subunit was found in about 7% of TH-positive cells, and also on a previous report according to which DA cells can express the GABA_A_R α1 subunit mRNA [51]. Using our sensitive immunofluorescence protocol, we also found that α3-positive clusters are present in both DA and non-DA cells and that the α1 and α3 subunits can be co-localized at the same synaptic hot spots (Fig. 1F,G). These results reveal a complex organization of GABA_A_Rs in VTA neurons and may help explain why GABAergic currents are reduced, but not abolished, in midbrain DA cells of α3 knockout mice [52].

Previous studies have shown that GABA_A_Rs are more densely located in non-DA cells than in DA neurons of the VTA [23], [53], [54], and that activation of GABA_A_Rs in GABAergic cells causes an increase of mesolimbic dopamine levels by relieving the inhibitory, GABAergic input to DA neurons [23], [27], [31], [53], [54]. The differential expression of GABA_A_Rs in DA and non-DA cells may explain the biphasic effects mediated by muscimol on the release of DA. In fact, low doses of muscimol preferentially inhibit GABAergic interneurons, resulting in disinhibition of DA cells, whereas higher concentrations of muscimol inhibit DA neurons [10], [23], [53]. These effects can be explained by the predominant expression of α1-GABA_A_Rs in non-DA cells (ref. [31] and this study), as well as by the differential GABAergic innervation patterns of DA and non-DA cells (Fig. 3). Indeed, we found a significantly higher density of GABAergic contacts in the dendritic domains of α1-positive non-DA cells compared to DA neurons. However, the density of perisomatic GABAergic synapes was similar in the two populations of neurons. This may explain why the frequency of mIPSCs was similar in GABAergic and DA cells [31]. The largely differential expression of the α1 and α3 subunits in DA and non-DA cells is predicted to affect the functional properties of GABAergic inhibition. Indeed, Tan and colleagues [31] reported that mIPSCs in VTA GABAergic neurons were slower and bigger than those in DA neurons. This is surprising, however, because α1-GABA_A_Rs are generally characterized by fast decay kinetics that ensure fast synaptic inhibition [55]–[57]. It is possible that additional factors, such as the co-expression of α1 and α3 subunits (Fig. 1F,G) or the geometry of synaptic appositions [58], may contribute to explain the unusual kinetics of α1-GABA_A_Rs in non-DA cells. Another possibility is that the data obtained by Tan et al. [31] were mainly derived from the subgroup of non-DA neurons that have undetectable levels of the α1 subunit (Table 2).

Another finding of the present study is the strong expression of the GABA_B_R1 subunit in both DA and non-DA cells of the rat VTA (Fig. 4). The expression of GABA_B_R1 was particularly prominent in DA cells, that exhibited GABA_B_R1 labeling in both the somatic and dendritic compartment. This may be related to previous observations according to which the majority of GABA_B_Rs are located on DA neurons [10], [59]. However, further studies have shown that GABA_B_Rs are expressed in both DA and GABA neurons, although different GABA_B_R agonists exert opposing effects on the reward system (see ref. [60] and references therein). This differential effect is due to the much weaker coupling between GABA_B_Rs and a G protein-gated, inwardly rectifying potassium channel in DA cells compared to non-DA cells [60], [61].

Because VTA interneurons provide inhibition to DA cells, they are believed to play a crucial role in the control of DA release and in the changes in VTA circuits that underlie addictive behaviors. The present study contributes to our understanding of the anatomy and neurochemical organization of GABAergic microcircuits of the VTA and lays the groundwork for future studies examining the plasticity of GABAergic circuits induced by drug exposure.
